# Monodisperse Gold Nanoparticles: A Review on Synthesis and Their Application in Modern Medicine

**DOI:** 10.3390/ijms23137400

**Published:** 2022-07-02

**Authors:** Mohammed Ali Dheyab, Azlan Abdul Aziz, Pegah Moradi Khaniabadi, Mahmood S. Jameel, Nazila Oladzadabbasabadi, Selwan Abduljabbar Mohammed, Raja Saleh Abdullah, Baharak Mehrdel

**Affiliations:** 1 School of Physics, Universiti Sains Malaysia, Gelugor 11800, Malaysia; shokryy8844@gmail.com (M.S.J.); aldobaselwan@gmail.com (S.A.M.); rajasaleha@gmail.com (R.S.A.); 2 Nano-Biotechnology Research and Innovation (NanoBRI), Institute for Research in Molecular Medicine (INFORMM), Universiti Sains Malaysia, Gelugor 11800, Malaysia; 3 Department of Radiology and Molecular Imaging, College of Medicine and Health Science, Sultan Qaboos University, Muscat 112, Oman; pegah32121065@gmail.com; 4 Food Biopolymer Research Group, Food Technology Division, School of Industrial Technology, Universiti Sains Malaysia, Gelugor 11800, Malaysia; oladzad.nazila@gmail.com; 5 Department of Physiology and Pharmacology, Thomas J. Long School of Pharmacy & Health Science, University of the Pacific, Stockton, CA 95211, USA; baharimehr@gmail.com

**Keywords:** monodisperse AuNPs, surface modification, imaging, therapy, theranostic

## Abstract

Gold nanoparticles (AuNPs) are becoming increasingly popular as drug carriers due to their unique properties such as size tenability, multivalency, low toxicity and biocompatibility. AuNPs have physical features that distinguish them from bulk materials, small molecules and other nanoscale particles. Their unique combination of characteristics is just now being fully realized in various biomedical applications. In this review, we focus on the research accomplishments and new opportunities in this field, and we describe the rising developments in the use of monodisperse AuNPs for diagnostic and therapeutic applications. This study addresses the key principles and the most recent published data, focusing on monodisperse AuNP synthesis, surface modifications, and future theranostic applications. Moving forward, we also consider the possible development of functionalized monodisperse AuNPs for theranostic applications based on these efforts. We anticipate that as research advances, flexible AuNPs will become a crucial platform for medical applications.

## 1. Introduction

Cancer is one of the leading causes of death worldwide [1]. It is distinguished by the uncontrollable growth of cells resulting from genetic alterations. The standard treatment is surgery, which is limited to tumors that are accessible. Chemotherapeutic medicines are also employed; however, their activity causes harmful side effects since it is not restricted to malignant cells. To avoid this issue, the medications could be selectively delivered into cancerous cells through vectors. As a result, the application of nanoscience in the medical field is increasing at an exponential rate [2]. Gold nanoparticles (AuNPs), iron oxide, dendrimers, liposomes, and polymers are the most commonly utilized nanoparticles in cancer nanotechnology [3]. AuNPs, in particular, have been the topic of extensive research recently. It has been demonstrated that they can identify cancer cells preferentially [4]. AuNPs in medical applications have sparked attention in recent decades due to their intrinsic features, which make them suited for cancer diagnosis and treatment. AuNPs, like many valuable metals, have a superior optical feature called surface plasmon resonance (SPR), which permits them to be employed in near-infrared (NIR) resonant medical imaging modalities, including computed tomography (CT) [5], X-ray scatter imaging [6], fluorescence imaging [7], photoacoustic imaging (PAI) [8] and MRI [9]. When subjected to NIR laser light, AuNPs generate heat, making them appropriate for photothermal cancer treatment [10,11]. Furthermore, AuNPs are nonimmunogenic and have low toxicity. The synthesis procedures of AuNPs are straightforward; therefore, the shape, size and surface modification are manageable. All of these qualities imply that AuNPs can be modified in a variety of ways for the localized hyperthermia of cancer tissue as well as regulated and targeted drug delivery [12]. AuNPs have been examined and delivered in clinical trials (phases I and II) for cancer treatment based on their excellent characteristics [13]. One of the essential factors of AuNPs that influences circulation half-life in the body, systemic toxicity, tumor accumulation, and other aspects important for diagnostic and therapeutic applications is size. As the number of applications for AuNPs expands, it is critical to better understand the fundamental impacts of AuNPs of varying sizes. Many factors influence the fluorescence of AuNPs, including size, surrounding environment, oxidation state and surface chemistry. Furthermore, it was discovered that the catalytic efficiency of AuNPs differs significantly from that of nanoclusters [14,15]. The oxidation of organic free radicals to stable molecules has profound implications for cell biochemistry. To begin with, it repairs radiation damage, which prevents molecules from being attacked by free radicals such as ˙OH from being fixed by antioxidants and other cell healing mechanisms. Furthermore, if some of the molecules generated in cells by radiation are signal molecules (i.e., molecules that initiate cellular apoptosis), their enhanced production owing to redox catalysis will result in greater cell death. Capturing the unpaired electron of an organic radical by NPs, on the other hand, can have a positive effect, such as preventing radical chain processes such as lipid peroxidation in cell membranes [16].

A recent study found that biocompatible AuNPs had significant antibacterial activity, whereas bigger AuNPs are ineffective [17]. Furthermore, the improved biodegradability, renal clearance and pharmacokinetics of AuNPs have been thoroughly documented [18,19]. Monodisperse AuNPs hold great promise in biological applications due to their unique characteristics and natural biocompatibility [20,21]. The ability of AuNPs to enter the nucleus was also investigated. Huang et al. and Kumar et al. created AuNPs with diameters of 6 nm and 2 nm, respectively, and discovered that these NPs can successfully penetrate the nucleus [22,23]. Furthermore, smaller AuNPs are less hazardous than larger AuNPs. According to several research studies, AuNPs can finally be eliminated from the body via the glomerular filtration system, rather than collecting extensively in various organs, tissues and cells [24,25,26]. Zheng et al. demonstrated that particle size affects renal clearance efficiency, and AuNPs with a size of about 6 nm could be removed from the blood through filtration by the kidney to the bladder [27]. Stevens et al. established indirectly that monodisperse ultrasmall AuNPs may be entirely removed from the body via the kidney and liver by constructing an AuNP disease detection platform [28]. As a result, the intrinsic features of monodisperse AuNPs increase their potential for the diagnosis and therapy of cancer. Monodisperse AuNPs have made remarkable progress in disease detection and therapy as a revolutionary nanomedicine, and there is an urgent need for a comprehensive review of monodisperse AuNPs and their theranostic applications.

Mathilde et al. [29] summarized gold nanoparticles for molecular imaging in cells and living systems, whereas Alizadeh et al. [30] discussed AuNP aggregation applications. Halawa et al. [31] described the synthetic strategies and recent advances in fluorescent sensing with AuNPs. None of these studies focused on the methods for producing monodisperse AuNPs and their theranostic applications.

Despite numerous earlier reviews on the use of AuNPs in medical applications, this is the first report on the synthesis of monodisperse AuNPs and their prospective use in cancer diagnostics and therapy (theranostic applications).

In this review, we will concentrate on monodisperse AuNPs synthesized using diverse methods for various diagnostic and therapeutic applications. The production methods, type, shape, properties, and surface modifications of monodisperse AuNPs will be discussed initially. The usage of monodisperse AuNPs in cancer diagnostic imaging techniques such as optical imaging, photoacoustic imaging, fluorescence imaging, CT and MRI will be briefly summarized. The applications of monodisperse AuNPs in cancer therapy, including photothermal therapy, photodynamic therapy, radiotherapy, chemotherapy and drug delivery will be discussed in more detail. This information could be useful in developing clinical uses for AuNPs in the future.

## 2. Monodisperse AuNPs

**Figure 1:**
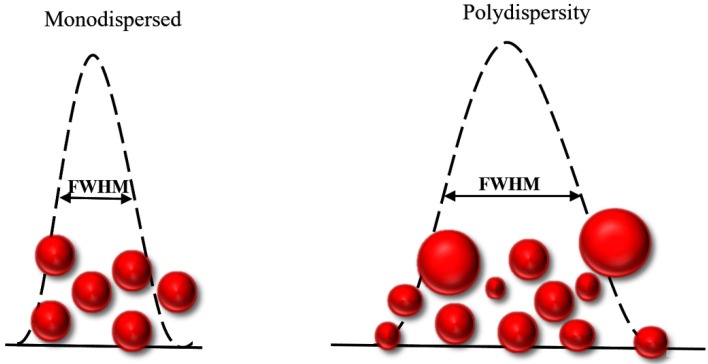
Concept of narrow and width size distribution. Low FWHM indicates monodisperse AuNPs and higher values indicate polydispersity of AuNPs.

Colloidal dispersions of AuNPs based on the variety of their dimensions are generally polydisperse. However, researchers tend to frequently use monodisperse nanoparticle systems as a model with narrow variation of AuNP size distribution. Generally, AuNPs’ size indicates a number average. The size of AuNPs can be determined by measuring the diameter of a large number of unique particles, employing different techniques, for instance, electron microscopy, light scattering and analyzing data collected by X-ray diffraction. Monodisperse AuNPs with a size variation of less than 5.0% (relative standard deviation less than 5.0%) show unique properties and exhibit a higher performance compared to polydisperse AuNPs [32]. Monodisperse colloidal AuNPs with controlled morphologies, size distribution, surface chemistry, and optical properties show a high potential for use in biomedicine, rapid identification, and the imaging of cancer cells in a living state. Hence, the production of monodisperse AuNPs is critical in the design of NPs for their specific applications. For instance, the AuNPs employed for sensing applications with a narrower plasmonic peak and a smaller full width half maximum (FWHM) are preferable for achieving a higher sensitivity and a lower limit of detection in studies (Figure 1) [33,34]. Moreover, developing a controllable, time-saving and convenient method to prepare monodisperse AuNPs with tunable plasmonic spectra and high uniformity is receiving tremendous attention. Recently, Jairo et al. [35] demonstrated the importance of fractional design in creating precise AuNPs employing sodium borohydride (SB) and sodium citrate (SC) as reducers. The suggested method employs simple and low-cost ways to produce monodisperse AuNPs without the need of time-consuming image-based characterization equipment. In this research work, FWHM as a function of AuNPs’ plasmonic band was employed as a variable of reaction [35].

**Figure 2:**
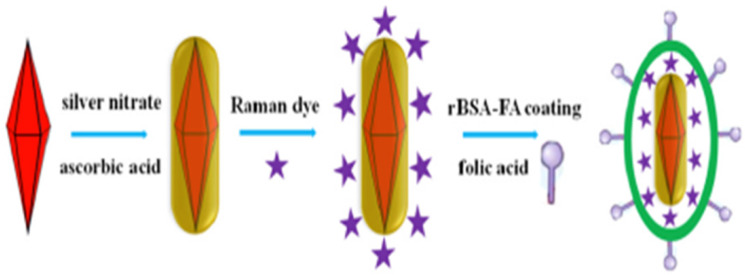
Illustration of fabrication of AuNBP@Ag NR-MBA-rBSA-FA nanoprobes [33]. Copyright Elsevier 2019.

In addition, the synthesis of uniform-sized and highly monodisperse AuNPs is possible by utilizing the seeding growth method, in which tiny AuNPs are initially produced and then employed as a nucleation center for the formation of bigger NPs [36]. Moshaii and his colleagues studied the dependency of polydispersity and size on the reducing agents’ concentrations. Their findings showed that monodispersed AuNPs (with a size range of 2.5 to 35 nm) can be synthesized in particular ranges of reducing agent concentrations [36]. Cui and his group reported a time-saving and straightforward approach for synthesizing monodisperse Au nanobipyramid core and silver nanorod shell NPs (Au NBP@Ag NRs). The Ag shell directly grows on the surface of the Au NBP core (Figure 2). In this work, the authors reported Au NBP with a highly uniform size distribution (with a relative standard deviation of less than 5.0%) and the average size for Au NBPs was 80 nm (length) and 50 nm (width) with high stability. In addition, the TEM and SEM images showed Au NBP@Ag with a length of 100 nm and width of 50 nm. Their proposed monodisperse core shell NPs have a number of distinct benefits, including good responsivity, high stability, favorable homogeneity and high Surface-Enhanced Raman Resonance (SERS) activity. Proposed Au NBP@AgNRs are capable of promptly identifying live cancer cell cultures without additional therapy [33].

Xu et al. demonstrated the ultrasensitivity of highly uniform and monodispersed Au NBPs for colorimetric biosensing. They developed an ultrasensitive colorimetric immunoassay for alkaline phosphatase (ALP) activity based on the targeted guided deposition of Ag on extremely uniform and monodisperse Au NBPs [37,38]. It is believed that protocols developed for the preparation of monodisperse colloidal AuNPs with a very narrow size distribution will offer versatile coupling points for several bioconjugation-based applications (e.g., bioprobes and drug deliver) [39,40]. Han et al. [39] reported a fast and facile synthetic route to synthyze monodisperse Au@SiO_2_ NPs (50 nm Au cores and ~35, 75 and 90 nm silica shell thicknesses) with a pure silica surface for functionalization, without any specific interface to enable bioconjugation via well-established silica surface chemistry.

In addition, the preparation and design of polymer-functionalized monodisperse colloidal AuNPs for drug delivery have attracted interest due to their higher biocompatibility, stability, and controlled release of drug [41]. For instance, Venkatsan et al. reported a developed method for AuNRs-doxorubicin conjugation by an electrostatic interaction between the amine group (−NH_2_) of DOX and the negatively charged PSS-AuNRs (AuNRs coated with poly sodium 4-styrenesulfonate) surface. The improved monodispersed AuNRs (4.4 aspect ratio) exhibited a high efficiency in drug loading and higher biological stability compared to free DOX AuNRs [42]. Thus, monodisperse AuNPs with a narrow size distribution will open up a new path for the development of revolutionary noble AuNP growth-based biosensors and various biomedical applications [43].

## 3. Synthesis of Monodisperse AuNPs

**Figure 3:**
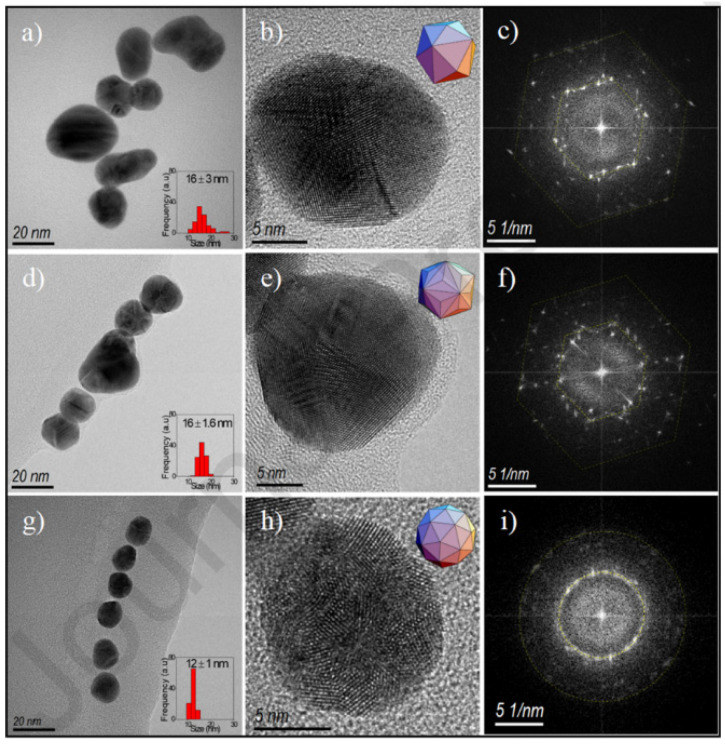
The TEM images of AuNPs are shown in the first column—(**a**,**d**,**g**)—with graphs of size distribution shown in the insets. The HRTEM images in the second column—(**b**,**e**,**h**)—are polyhedral insets. We can see the SAEDs in the third column—(**c**,**f**,**i**) [60]. Copyright Elsevier 2021.

Because of the possibilities of AuNPs in diagnosis and therapy, AuNP synthesis has been extensively studied for centuries. The present research has confirmed tremendous progress in the preparation of AuNPs. AuNPs can be synthesized in a variety of ways, using physical and chemical methods. More specifically, these production methods can be divided into two categories: top-down and bottom-up methods [44]. To reach nanoscale structures, top-down approaches usually use bulk materials as well as removal strategies, including lithography. On the other hand, bottom-up approaches rely on the unit coordination of material molecules for nanoparticle synthesis by monitoring the growing structure. Sonochemical, thermal reduction, and electrochemical procedures are examples of bottom-up methods [5,45]. The most popular bottom-up methodologies used for the production of AuNPs are the Brust and Turkevich techniques. The Turkevich technique depends on reducing metallic ions to generate spherical and homogeneous AuNPs with dimensions varying from 10 up to 20 nm [45]. Sodium citrate is generally used as a reducing agent as well as a stabilizer, resulting in a colloidal dispersion that prevents particle aggregation [46]. Furthermore, instead of citrate, UV light, ascorbic acid, and amino acids can be used [47]. The Schiffrin–Brust approach, which is premised on multiple procedures that are advantageous for the synthesis of AuNPs in an organic system with high stability, was first revealed in 1994 [45]. This method utilizes tetrabutylammonium bromide as a transfer agent from organic to inorganic solutions, and particle sizes ranging from 2 up to 6 nm can be acquired [48]. Aside from these approaches, the “growing seed” technique is commonly used to prepare other shapes of AuNPs, such as nanocubes and nanorods [49,50]. In this method, tiny seeds are synthesized first, serving as nucleation centers. Under controlled conditions, the reactive sites on all of these nucleation centers can then grow to ensure AuNPs of a predicted size and with the desired shape. Two reducing agents usually used in this method are hydroxylamine and ascorbic acid. The size of the particles produced is determined by the Au ion ratio, which ranges from 5 to 40 nm [51]. The radiation method has been effective in nanomaterial research [52] (Table 1), since it has been investigated primarily using high-energy charged particles, such as ions and electrons, and also photons such as X-rays and gamma rays [53]. Nonionizing radiation sources including microwave and ultraviolet (UV) light at certain wavelengths. Enzymes [54], nanocomposites [55], metal nanoparticles [56], hybrid nanoparticles [57], and organic nanoparticles such as proteins [58] are examples of nanostructures that can be designed and synthesized using radiation. Nguyen et al. [59] reported the synthesis of homogeneous AuNPs with sizes ranging from 5 to 40 nm via the radiation method. The results showed that the irradiation process is suitable for the synthesis of AuNPs with high purity and a controllable size. The sonochemical method which is considered as rapid, easy, cost-effective, attractive, and eco-friendly has been reported to synthesize AuNPs. Fuentes-García et al. synthesized AuNPs using different ultrasound irradiations (60, 150, and 210 W). Colloidal AuNP solutions were acquired from gold acid (HAuCl_4_) and sodium citrate for 60 min under irradiation [60]. Figure 3 depicts representative TEM images of AuNPs. The 60 W sample had a particle size of about 16 nm. The crystalline structure of AuNPs was characterized by HRTEM as a hexahedron polyhedral arrangement (Figure 3b), and the complex array was confirmed by the selected area electron diffraction (SAED) pattern (Figure 3c). The triakis icosahedron crystallization was visible in the 150 sample (Figure 3d). However, there were no discernible changes in particle size (Figure 3e). The size was reduced to 121 nm in the 210 sample (Figure 3g). The rings in the simulated electron diffraction patterns image confirmed a quasi-spherical shape as a faceted pentakis dodecahedron (Figure 3h,i).

Various methods for producing AuNPs that have recently been introduced in order to identify physicochemical features will be thoroughly explored. These methods are: the (i) Turkevich, (ii) Brust, (iii) seed-mediated growth, (iv) biological synthesis and (v) sonochemical methods.

### 3.1. Turkevich Method

The Turkevich method for producing AuNPs was initially described in 1951. It is one of the most widely utilized procedures for producing spherical AuNPs. This approach produces AuNPs with sizes ranging from 1 to 20 nm, based on conditions [61]. The main idea behind this approach is to reduce gold ions to form gold atoms via reducing agents such as citrate [62], ascorbic acid [63], and amino acids [64]. The stabilization of AuNPs is accomplished by the use of several capping/stabilizing agents. Initially, the Turkevich method’s uses were limited due to the limited variety of AuNPs that could be produced using this process. Several developments in the fundamental process have allowed researchers to broaden the size range of the particles generated using this technology over time. It was discovered in 1973 that by adjusting the ratio of reducing and stabilizing agents, AuNPs with specific sizes ranging from 16 to 147 nm could be generated [65].

### 3.2. The Brust Method

This approach, which was first published in 1994, uses a two-phase procedure to synthesize AuNPs with sizes ranging from 1.5 to 5.2 nm by utilizing organic solvents [66]. The process entails the employment of a phase transfer agent, such as tetraocty lammonium bromide, to transfer Au ions from their aqueous solution to an organic solvent such as toluene. The Au is subsequently reduced using a reducing agent including sodium borohydride in conjunction with an alkanethiol. The alkanethiol is responsible for the stability of AuNPs [67]. The color shifts from orange to brown as a result of interaction [68].

### 3.3. Seed-Mediated Growth

The previous two approaches can produce only spherical AuNPs; however, they can also be formulated in a number of shapes and geometries such as rods [69]. Seed-mediated growth is the most extensively utilized method for producing rod-shaped AuNPs. This process is based on the basic premise of first generating seed particles through reducing gold ions. This process is carried out in the presence of reducing agents such as NaBH_4_. The seed particles are then transferred to metal ions and a mild reducing agent such as ascorbic acid, which prevents additional nucleation and accelerates the production of rod-shaped AuNPs. The geometry and shape of AuNPs are determined by the seeds and concentration of reducing agents.

### 3.4. Biological Synthesis

Although chemical procedures are the most widely utilized method for producing metallic nanoparticles, the employment of costly and hazardous chemicals as stabilizing and reducing agents limits their usage. Furthermore, these nanoparticles may be toxic in biomedical applications [70]. As a result, there is an increasing need to develop cost-effective and environmentally friendly techniques for the production of nanoparticles that do not rely on toxic chemicals. In recent years, the biological production of nanoparticles has received a lot of interest as an eco-friendly and green method. Simple bacterial cells to sophisticated eukaryotes are among the biological resources employed in NP manufacturing. Surprisingly, the ability of organisms to fabricate metal nanoparticles has resulted in a new and exciting approach to the design of these biological nanofactories [71]. A wide range of species, including fungus, algae, plants, and bacteria, have been found to successfully synthesize AuNPs.

### 3.5. Sonochemical

Sonochemistry facilitates the synthesis of monodisperse AuNPs by providing a unique crystallinity control [72]. Sonochemical methods are fairly inexpensive in comparison to most techniques, allowing researchers to more freely experiment and explore ideas [73]. Sonochemical synthesis requires less than 30 min for production, compared to solvothermal methods, which take roughly 48 h. Additionally, the particles produced by sonochemical methods are monodisperse and smaller in size than those produced by traditional synthesis [74]. Sonochemical processes are derived from the strong transient conditions generated by ultrasound, which generates different hot spots with temperatures of about 5000 K, cooling and heating rates of up to 10^10^ K s^−1^ and pressures over 1000 atm [9,75]. These conditions differ from other conventional synthetic processes, such as photochemistry, wet chemistry, flame pyrolysis and hydrothermal methods [76], which do not have the same conditions. Based on periodic expansion and compression, ultrasonic waves flow through a typical liquid from low- and high-pressure zones [77]. This pressure change leads to the start of sonochemistry, which occurs before the crucial period of sonic cavitation, i.e., the creation, expansion, and bubble collapse. The action of bubble expansion and compression continues until external pressure triumphs and the bubble explodes. These conditions can cause anomalous chemical and physical changes, as well as enhance a specific reaction between atoms and molecules, resulting in the formation of a new class of materials [78]. Nevertheless, the value of the sonochemical process stems from the fact that the radicals and ions inside the bubble are emitted by chemical solutions; hence, appropriate chemicals will aid in modifying the overall method. These conditions allow for the sonochemical synthesis of several nanomaterials.

The sonochemical production of monodisperse AuNPs has great potential. This technique is a relatively simple and powerful method for generating nanomaterials, and it is able to control the properties of AuNPs by adjusting the ultrasonic process parameters [79,80]. Monodisperse AuNPs with spherical shapes and an average size of around 18.5 were synthesized using an inexpensive sonochemical approach in which the nanoparticles were generated using ultrasound from droplets of the metal salt precursor solution.

### 3.6. Advantages and Limitations of the Methods

The Turkevich method is a simple and repeatable procedure for producing spherical AuNPs with sizes ranging from 10 to 30 nm. However, once NPs’ sizes exceed 30 nm, they will be less spherical in shape and have a greater size distribution. Furthermore, this process has a low yield and uses only water as the solvent [81]. The Brust approach, on the other hand, entails a simple strategy for producing thermal as well as air-stable AuNPs with a controlled size and low dispersity. One potential restriction of the Brust approach is the production of AuNPs that are less distributed and employ organic solvents that are immiscible with water, inhibiting their biological applicability [48]. Seed-mediated growth is a viable approach for producing rod-shaped AuNPs, but different parameters influence rod size and should be carefully managed. In one study, higher HAuCl_4_ concentrations resulted in larger seed rods with lower aspect ratios. Temperature also performs an important role in the formation of rods, with higher temperatures producing rods with lower aspect ratios. In order to accelerate rod growth, a number of seeds introduced to the mixture should be carefully examined [82]. Furthermore, chemical approaches have their own set of limitations, which also include biocompatibility and environmental considerations. A few of the chemicals utilized in the fabrication of AuNPs during chemical esis can harm our environment and pose risks when administered to living organisms, restricting the biological applications of these NPs [83]. To address these problems, numerous biological approaches for the preparation of AuNPs have been developed.

The synthesis reaction of AuNPs, carried out by taking a biological approach, can take hours or even days. The preparation of AuNPs from plant-based materials is a simple and straightforward procedure. The reaction parameters can be used to control many aspects of AuNPs including size and shape. Furthermore, the reaction time is short and monotonous. The limitations of employing plants for AuNP formation is that it is difficult to identify reactive components since plant biomass contains a wide range of organic components [84,85,86].

The sonochemical approach is an environmentally friendly, green, rapid, and simple method of producing monodisperse AuNPs [87], and it has also been used successfully with less volatile organic liquids. Several types of nanostructures of oxides, metals and carbides can be created by varying reaction conditions [76]. A reducing agent is not required for the reduction of noble metal salts during nanostructure development, the reaction rate is generally quick, and very small metal particles are formed. The disadvantage of sonochemical reduction is that the rate of reduction is entirely dependent on the ultrasonic frequency [76].

## 4. Type of AuNPs

**Figure 4:**
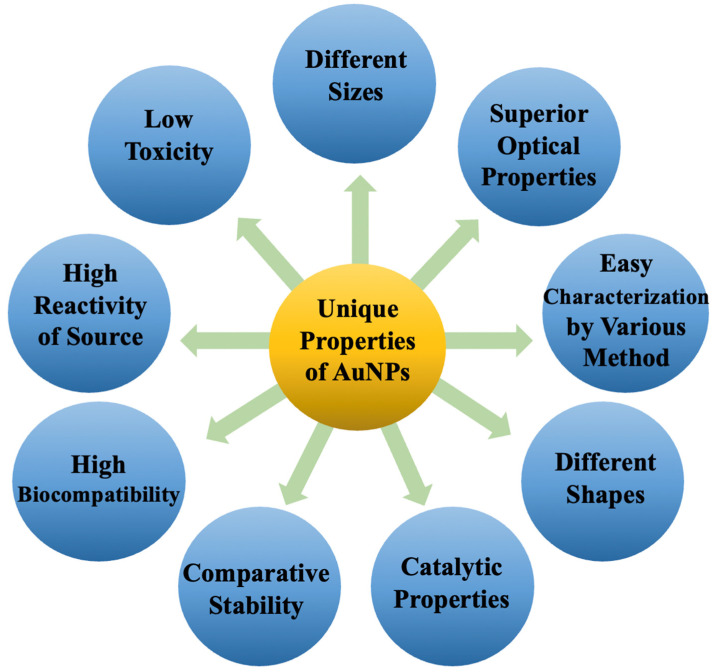
Unique properties of AuNPs.

AuNPs are typically produced by reducing a gold salt and coating it with an organic [90] or inorganic [91] layer to ensure colloidal stability. Surface plasmon resonance (SPR) is a property of AuNPs that is caused by the collective oscillations of free electrons along the gold lattices in the AuNP core [92]. Using different sizes, shapes, surface coatings, and assemblies of AuNPs, this SPR can be fine-tuned to obtain the desired optical properties (Figure 4). Since Turkevich’s seminal work on spherical AuNP synthesis [93], a plethora of adapted procedures have been developed that alter the process parameters to vary the sizes of spherical AuNPs [88,89]. Because of the greater surface-to-core ratio available in the conjugation of the dyes or targeting moieties, modifying AuNPs’ size is of specific importance for molecular imaging. Furthermore, increasing AuNPs’ size causes a bathochromic shift in their SPR, which is advantageous because several optical biomedical techniques are used in the near-infrared region (NIR), i.e., 650–900 nm, where tissue absorption is low [94]. On the contrary, the surface-to-core ratio has no impact on CT contrast enhancement, but it does affect the biodistribution and pharmacokinetics of AuNPs, allowing them to be used in a variety of molecular imaging applications [95].

## 5. Shapes of AuNPs

**Figure 5:**
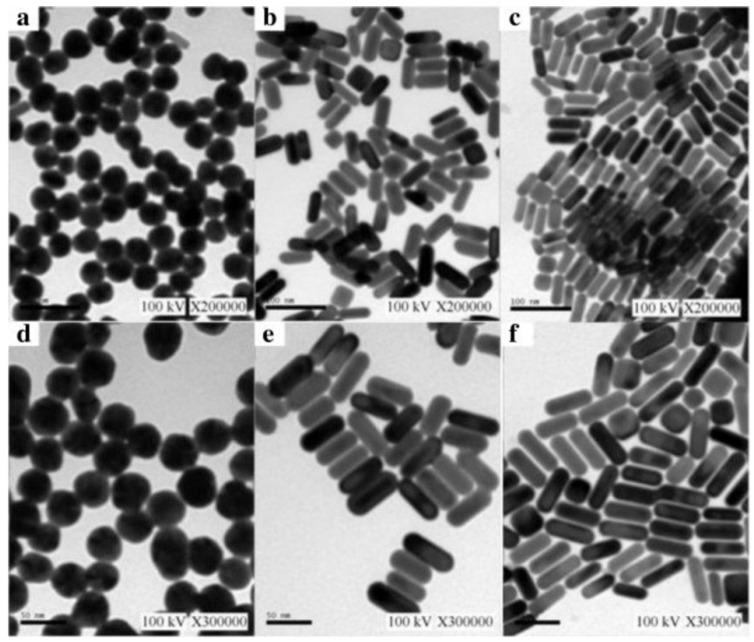
Various types of monodisperse AuNPs: (**a**) Au nanobones, (**b**) Au nanohoneycombs, (**c**) mesoporous silica-coated Au nanorods, (**d**) Au nanocages, (**e**) Au nanorods and (**f**) Au nanospheres. Adapted from [104].

Several methods for synthesizing AuNPs with various shapes have been developed. For instance, gold nanorods (AuNR) have both longitudinal and transverse SPRs, which can be shifted to the NIR region by modifying the length-to-width ratio [96]. Surprisingly, miniature AuNRs with SPRs in the NIR-II, i.e., beyond 900 nm, have been reported [97]. When compared to larger AuNRs, these miniature AuNRs allowed for a 30% advancement in tumor accumulation and an around 4.5-fold improvement in photoacoustic imaging (PAI) contrast in tumor imaging. Core@shell NPs were also established as effective contrast agents for a variety of modalities as well as cancer treatments [98]. Notably, a sphere-shaped iron oxide core was coated by a gold shell that provides T_2_ contrast in MRI, allowing CT and MRI multimodal imaging [99]. Other unique-shape variations, such as stars, triangles, and prisms, have been formed to adjust both the cellular uptake and optical properties of AuNPs (Figure 5) [100]. Gold nanoprisms’ strong light scattering characteristics make them suitable contrast agents for skin imaging or the imaging of melanoma tumors using optical coherence tomography (OCT) [101]. Stars have a high surface-to-core ratio, which is beneficial for the surface modification of targeting ligands and allows for the molecular imaging of CT [102] and SERS [103].

## 6. Properties of AuNPs

**Figure 6:**
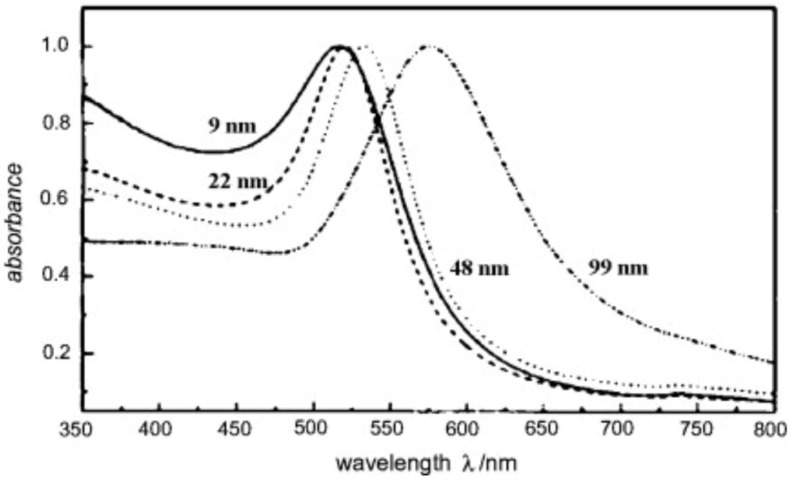
The UV–visible spectra of AuNPs with different sizes [107]. Copyright Elsevier 2010.

Surface plasmon resonance (SPR) is one of the common essential features of AuNPs, and it is mostly determined by their shape and size [105]. Plasmon is a collective oscillation of the free electrons at the material interface that happens when certain wavelengths of light interact with the conduction band of the material interface, causing a dipole oscillation that relies on the electromagnetic field and ionic lattice of incident light. The maximum oscillation happens at a certain light frequency, which is known as SPR [106]. AuNPs absorb light strongly depending on their size, and the SPR band spans the visible to infrared region. Figure 6 depicts the displacement of SPR peaks caused by different AuNP sizes [107]. SPR also offers surface plasmon scattering, which occurs when light strikes AuNPs and causes electron oscillation, which results from photon energy and re-emits photons of the same wavelength. Importantly, altering the material interface with various receptors and the consequent interaction with diverse structures such as cells affect the wavelength, which can be exploited for the imaging and diagnosis of various cancers such as breast, prostate and lung cancers [108]. Furthermore, AuNPs can emit the previously mentioned light which induces collective oscillation, resulting in heat, making them a suitable platform for various therapeutics including cancer cell eradication via photothermal therapy and drug-delivery systems. When the bulk size of gold is reduced to nanoscale dimensions, the surface-to-volume ratio will increase, which impacts AuNPs’ surface energy and improves atom alignment on the nanoparticle surface. As a result, AuNPs have the ability to interact with many types of compounds in order to increase their biocompatibility [109].

## 7. Surface Modification

AuNPs are simple to functionalize and can interact with a variety of molecules, which allows one to manipulate AuNPs’ functions by conjugating biological molecules and chemical groups such as DNA, antibodies and peptides for various purposes, including diagnostic cancer therapy and gene-/drug-delivery systems [110]. Generally, these ligands allow nanoparticles to reach the desired region and perform detection or therapeutic functions. Physical and chemical interactions between ligands and AuNPs vary depending on the ligand type and synthesis method [111]. Covalent bonding has received more attention in chemical conjugation than other techniques due to its efficient nature and excellent stability in a physiological environment. Additionally, the size of modified AuNPs can be controlled more precisely, and the coupling between AuNPs and molecules is stable. This technology is simple to use, but the number of available and suitable ligands for this technique is restricted. Ligand exchange considers a very novel method of conjugating ligands onto the surfaces of AuNPs, wherein hydrophobic ligands interchange with hydrophilic ones to offer preferred surface features [112]. In this approach, the first reactive group of a functional chemical binds to the surface of the AuNPs, while the other functional group of the compound serves as a coupling site for other molecules including peptides and antibodies. Trialkoxysilane is commonly utilized in this approach due to the number of coupling sites that can connect with other ligands, particularly those with vinyl and amino groups. Phosphine’s interaction with AuNPs is very weak; thus, it is an excellent option for exchanging with thiol groups to further modify them to improve particle stability in physiological environments. The effectiveness of this approach is determined by a number of parameters, such as ligand type, nanoparticle size, bonding strength and the number of grafted ligands [111].

The surface modification of AuNPs is required to reduce surfactant-induced toxicity and improve biocompatibility. The thiol gold reaction, which relies on the high affinity between Au and thiols, is the most essential approach for functionalizing AuNPs. By replacing the CTAB with thiolated species, AuNPs using hexadecyl trimethyl ammonium bromide (CTAB) as a surfactant could be detoxed and stably dispersed [113,114]. Liang et al. used gold–sulfur bonding to produce stably dispersed AuNPs with different sizes, about 2, 4, and 6 nm, covered with zwitterionic ligands [115], whilst Garcia et al. [116] generated AuNPs at a size of about 5 nm stabilized with double-pyridine salt in cancer treatment. Wu et al. [117] synthesized AuNPs with a size of about 12 nm with folic acid and the reduction of a bovine serum albumin conjugation for imaging and photothermal treatment. The thiol gold reaction is also used in various methods for the surface modification of AuNPs, such as electrostatic adsorption. Rotello et al. [118] created a DNA-delivery system through electrostatic interactions, combining DNA with trimethylammonium mixed monolayer protection cluster-adjusted AuNPs. Rotello et al. [119] produced a gold core of 2 nm and triethylenetetramine-terminated ligands that electrostatically reacted with negatively charged siRNA. The versatility of the gold–sulfur bond serves as the foundation for the surface modification of AuNPs, expanding the potential for using AuNPs as a diverse cancer theranostic platform.

## 8. Imaging Applications of AuNPs

**Figure 7:**
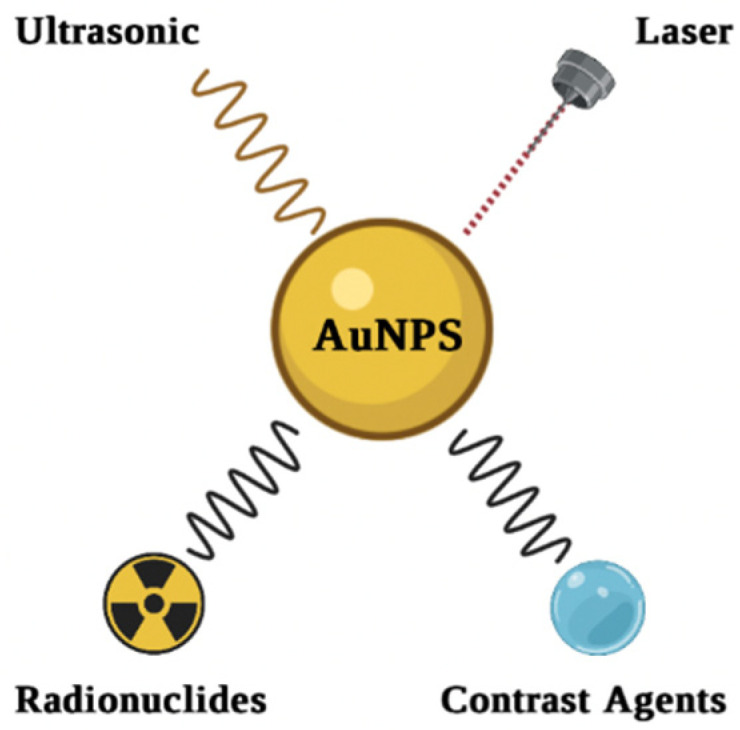
Imaging application of AuNPs.

Cancer is one of the major causes of death worldwide. Patients’ chances of survival are mostly dependent on diagnosing cancer in its early stages, when it is curable. Furthermore, present cancer diagnostic approaches are time-consuming, costly and have harmful side effects. Nanotechnology has improved diagnostic procedures in recent decades, allowing for the detection of cell alterations and cancer in its early stages [120,121]. AuNPs are considered the most viable candidate for imaging applications (Figure 7). To detect cancer cells precisely, a bioimaging system must have high selectivity and sensitivity. To prevent clearance, AuNPs must be functionalized in order to reach specific malignant cells while remaining undetectable to the immune system. PEGylated AuNPs, for example, covalently modified with a monoclonal antibody and herceptin (HER), can be bound to the targeted antigen on breast cancer cells [122]. The visible light absorption of AuNPs, which is caused by the SPR effect, is a useful characteristic for the detection of cancer cells using a colorimetric test. The chemical-physical properties of AuNPs, such as size, shape and solvent, have a direct effect on the SPR band of AuNPs. Furthermore, when the size of GNPs decreases, the wavelength switches from blue to red [52,90,91].

### 8.1. Optical Imaging

Up to now, researchers have employed AuNPs and several analytical optical imaging methods such as: (1) two-photon luminescence; (2) dark-field light scattering; (3) optical coherent tomography; (4) Raman spectroscopy; and (5) photoacoustic imaging for the optical imaging of cells and tissues [123,124]. A special imaging modality that can provide sectional images of a biological sample with a high resolution is optical coherence tomography (OCT). Gobin et al. [125] demonstrated that scattering is increased in the presence of Au nanoshells and that it can produce an improved optical contrast imaging for accurate tumor diagnosis in mice. Another imaging technology that allows for accurate cancer diagnosis in the early stages is photoacoustic imaging. This approach combines optical and ultrasonic imaging modalities. It is based on irradiating biological samples or tissues with short pulses of electromagnetic irradiation in the absorption range, which results in a raised temperature and local pressure, which can create measurable acoustic waves [124]. Eghtedari et al. [126] revealed that Au nanorods enhance the diagnostic power of laser photoacoustic imaging systems. They reported that Au nanocages provide more detailed images of vascular systems by increasing the contrast between blood and surrounding tissues by up to 81%. Additionally, it has been reported that nanocage-shaped AuNPs have larger optical absorption cross-sections than Au nanoshells and are more suited for in vivo applications [55].

Kang et al. [127] employed spherical AuNPs for fast subcellular Raman imaging to track changes in cell shape during toxic-induced cell death. High-resolution Raman images of different sites of the cell, including the cytoplasm, mitochondria, or nucleus, were obtained due to the good distribution of organelle-targeted AuNPs. Additionally, Ma et al. [128] performed a study on developed graphene oxide-wrapped AuNPs as a potential theranostic agent in HeLa cancer cells for intracellular Raman imaging (laser: 488 nm; strength: 20 mW) and doxorubicin administration. Using dark-field microscopy, Loo and colleagues [129] used the near-infrared light scattering of Au nanoshells as a contrast agent to identify the molecular marker human epidermal growth factor receptor 2 (HER2) inserted into breast cancer cells. Furthermore, Bickford et al. [130] showed that Au nanorods could potentially be usedas liver HER2-overexpressing cancer contrast agents for imaging using two-photon microscopy.

AuNPs were synthesized by Qian et al. [131] to be used as contrast agents in dark-field scattering light microscopy to analyze the life phases of cancer cells. Jin et al. [132] showed that DFM can be utilized to analyze carbohydrate–protein interactions using single plasmonic AuNPs [44].

### 8.2. Photoacoustic Imaging

In photoacoustic imaging (PAI), AuNPs can potentially be used as contrast agents. AuNPs have a large absorption cross-section which is greater than those of well-known chemical dyes such as rhodamine-6G and indocyanine green [133]. The intensity of the PAI signal is related to the concentration of AuNPs; increasing the concentration of AuNPs increases the PAI signal, which improves the image contrast [133]. Sentinel lymph node mapping is a method that can be used to successfully diagnose metastases in cancer staging. To this end, several imaging methods have been developed, the majority of which rely on dangerous radioactive tracers [134,135].

Han et al. [130] employed an epidermal growth factor receptor which conjugated to AuNPs with particle sizes ranging from 5 to 40 nm for cancer detection. The results revealed that the 5 nm AuNPs exhibited high near-infrared absorption while maintaining the same PA signal as the 40 nm AuNPs. The findings proved that AuNPs could be used as a PAI agent for cancer cell detection in vivo. Wang et al. [136] demonstrated that the graphene oxide@ AuNPs could be potentially used as bimodal agents for photothermal treatment and PAI for ovarian cancer. Recently, Salah et al. [137] presented a novel bio-imaging approach as well as a PAI agent utilizing PEG–CALNN–TAT Au nanorods.

### 8.3. Fluorescence Imaging

Fluorescence imaging (FI) is based on a linear connection between the intensity of the fluorescent signal generated by the stimulated fluorescent material and the amount of fluorescent substance present in a certain range. AuNPs have several well-known optical features and may passively accumulate at tumor locations, making them even more promising in early cancer detection than tiny therapeutic molecules. When utilized in tumor diagnosis, however, AuNPs accumulate abundantly in reticular endothelial system organs, lowering their targeting specificity and limiting their clinical applicability [138]. To effectively utilize the increased permeability and retention of tumors, AuNPs (≥10 nm) must be used since they may remain at greater concentrations in the plasma, therefore avoiding renal filtration [139]. Zheng et al. [140] applied FI to compare AuNPs (2.5 nm) coated with glutathione to the dye molecule IRDye 800CW in breast cancer tumor-bearing mice. The findings indicate that the use of gold NPs for tumor diagnosis was more efficient than IRDye 800CW. This finding supported the importance of renal-clearable ultrasmall fluorescent gold NPs in cancer diagnosis.

Hou et al. [141] demonstrated the tumor imaging capability of AuNPs using NIR BSA-Au nanoclusters as imaging contrast agents. Using HeLa and MDA-MB-45 tumor-bearing mice, ex vivo and in vivo investigations revealed that AuNPs were capable of accumulating in the tumor sites due to the EPR effects. Wang and colleagues employed GSH-capped silver nanoclusters as templates to create highly luminous AuNPs via a galvanic replacement approach [142]. The GSH-gold nanoclusters produced by Venkatesh et al. [143] can be employed as a possible purine-stabilized FI probe to identify CAL-27 cancer cells.

Likewise, the self-assembly of allylamine hydrochloride and GSH-AuNPs caused a significant increase in fluorescence via aggregation-induced emissions [144]. In vitro investigations revealed that the self-assembled nanocomposites had a much higher uptake than GSH-AuNPs. Liu et al. [145] developed a precision Au_25_(GSH)18 that fluoresces in the near-infrared region (1100–1350 nm) for the imaging of cerebral blood flow and brain cancer. Based on their in vivo findings, cerebral blood flow imaging was able to discriminate between healthy and damaged brains. Moreover, by utilizing Au_25_(GSH)18, primary cancer and lymphatic metastasis were diagnosed. Nanoparticle modification methods such as employing folic acid, an aptamer, a targeting peptide and a biosensor were functionalized on the surface of AuNPs to improve targeted cell imaging [146,147,148].

### 8.4. MRI

Magnetic resonance imaging (MRI) is a well-known imaging method based on the nuclear spin concept [149,150]. The use of MRI in the diagnosis of cancer is beneficial. Nonsystemic toxic AuNPs have great potential to be utilized in medical applications, such as being used as MR contrast agents [7]. Luo et al. [151] used AuNPs targeting prostate cancer in MR-guided radiotherapy to enhance targeting accuracy and effectiveness. The binding affinity and r1 relaxivity of AuNPs were greatly increased by conjugating gadolinium complexes and PSMA ligands to the surfaces of NPs. Elsewhere, the AuNPs bound with Gd significantly enhanced the image contrast of CT and MR images by using Gd as a contrast agent for MR imaging [152]. Another study by Cai et al. [101] investigated Au3 Cu1 nanoshells as agents for MR imaging blood vessels in in vivo studies, indicating their potential use as blood contrast agents in MR angiography. Due to their strong attenuation of CT and excellent MR signals, hybrid NPs with superparamagnetic iron oxide coated with AuNPs have been employed as dual contrast agents for CT and MRI [98,153]. Despite the limitations of Gd-based contrast agents in depicting tumor margins, gold nanoparticles have been used as contrast agents to detect brain tumors using MRI. Compared to Gd alone, Gd-conjugated AuNPs offer a substantially higher intracellular concentration of Gd and a longer-lasting amplification of brain tumor signals, leading to enhanced tumor imaging. As a result, a single preoperative injection of Gd-conjugated AuNPs allows for intraoperative MRI tumor excision, resulting in increased diagnostic accuracy and no toxicity associated with repeated doses of Gd chelates [154]. Iancu et al. [155] investigated nontoxic iron oxide@ AuNPs to reduce the T_2_ relaxation time in MR images of small animals. In another study, Wan et al. [105] developed a variety of T_2_ contrast agents using AuNPs and a dysprosium complex. The transverse relaxivity of AuNPs-DyDOTA(amide)2 was 22.9 mM^–1^ s^–1^ at 9.4 T after NMR spectroscopy, which is greater than the transverse relaxivity of dysprosium-based small molecules. Shahid [156] utilized a reduction process to synthesize water-based AuNPs stabilized by dimethylaminopyridine molecules. Based on this theory, butanethiol molecules are responsible for the limited tumbling of Gd-DTPA chelates, which induced a 38% enhancement in the relaxivity of AuNP-based MR contrast agents.

### 8.5. CT

CT is another frequent imaging modality in which AuNPs have been employed as a component that interacts with the weakening or amplified output signal, resulting in an enhancement of contrast in the CT images [5,157]. Most of the biocompatible particles are synthesized as an Au core with a water-soluble coating to increase the effectiveness of targeted CT contrast agents to specific cells/tissues [121,158]. Meir et al. [159] evaluated the migration, kinetics and distribution of T cells in vivo using AuNP-labeled melanoma-specific T cells and whole-body CT imaging. At the tumor region, the highest CT signal intensity was obtained 48 h after injection, revealing the accumulation of labeled AuNPs at the target, whereas the tumor region was not identifiable prior to injection. There was no visible CT signal for nontargeted T cells in the same specific region. Elsewhere, gold NPs have also been employed as cell-labeling contrast agents to test for monocyte accumulation inside plaques using CT, where the presence of AuNP-labeled monocytes in the aorta increased CT signal attenuation [160]. Cao et al. [161] demonstrated the fabrication of lactobionic acid (LA)-modified dendrimer-entrapped AuNPs as a specific hepatocellular carcinoma nanoprobe for using CT modality in vivo. Recently, Hara and colleagues [162] conjugated anti-prostate-specific membrane antigens (PSMAs) onto PEGylated AuNPs through (EDC/NHS) chemistry. PSMA-targeted gold NPs showed a significantly good performance as a contrast agent for the targeted CT imaging and X-ray fluorescence CT of prostate cancer.

### 8.6. PET

Positron emission tomography (PET), as one of the imaging modalities, can be used for quantitative imaging and has a high diagnostic potential for in vivo studies at the cellular and molecular levels. A radiometal such as ^64^Cu can be conjugated to gold NPs for radionuclide-based PET, in order to utilize the sensitivity of PET scans. Consequently, some studies have indicated that AuNPs can be used in the more sophisticated nuclear magnetic imaging method PET. Chen et al. [163] studied the performance of AuNPs (~2.5 nm) containing a ^64^Cu-labeled contrast agent to predict and diagnose kidney disease in vivo via PET imaging. Moreover, their findings proved the kidney’s quick clearance of GSH-coated AuNPs as well as the PET contrast agent for cancer detection and diagnosis.

The attachment of radiometal–chelator complexes meets significant difficulties due to the possibility of radiometal detachment as well as changes in the surface characteristics of AuNPs. A chelator-free ^64^Cu radiolabeling technique for PET imaging was developed to overcome these difficulties by chemically reducing ^64^Cu on the surface of RGD-PEG-Au nanorods (NRs) (50 × 15 nm). It was reported that RGD amino acid has a significant affinity to αvβ3 integrin receptors overexpressed on a variety of tumor cells, such as breast, bladder and prostate cancer cells, rendering it a unique molecular ligand for targeted cancer imaging/therapy [135,164]. Sun et al. [165] synthesized RGD-[^64^Cu]-PEG-Au NRs, and [^64^Cu]-PEG-AuNRs uptake in U87MG subcutaneous tumors was 8.37 1.16 percent ID/g (injected dosage per gram of tissue), and 6.19 0.5 percent ID/g at 24 h postinjection. In a U87MG glioblastoma xenograft model, these NRs have demonstrated high tumor-targeting capabilities and were effectively employed for PET image-guided photothermal treatment.

## 9. Therapy

**Figure 8:**
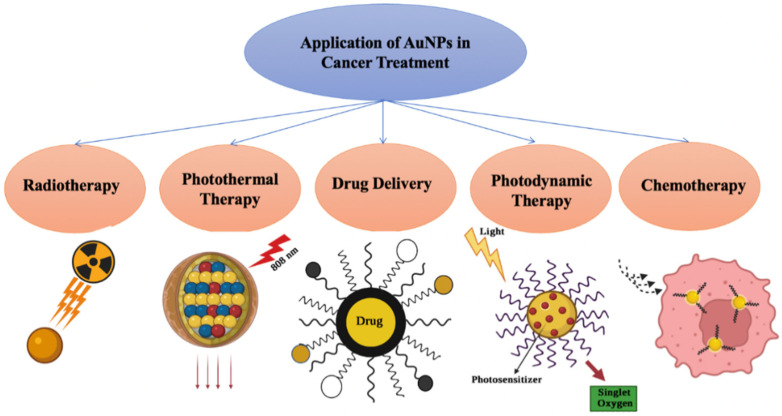
Various applications of AuNPs in cancer treatment.

Because of their unique optical as well as surface modification properties, AuNPs have enormous potential in cancer treatments such as PPT, PDT, radiotherapy, chemotherapy, and drug delivery, as explained in Figure 8.

### 9.1. Photothermal Therapy (PTT)

Because of the side effects of the current cancer treatment approaches, newer procedures such as PTT are being developed, which involve more minor damage to the healthy tissues [166]. PTT kills cancer cells by generating localized heat, which is most effective in the early stages of metastasis or when a tumor is in its initial stages [167]. PTT is a promising new cancer treatment approach that transforms light to heat and uses hyperthermia to trigger cell death [168,169]. When cells are cultured at temperatures higher than 42 °C for several minutes, permanent damage to the protein and membrane occurs, leading to cell death. The laser wavelengths and the penetration of light into the tissue are critical for the potency of PTT. Near-infrared (NIR) illumination is thought to be more capable of penetrating tissue [170]. AuNPs are considered one of the best options for this technique. Because of their unique optical properties, AuNPs may attract light at a certain wavelength (preferably and mostly in the NIR) and transfer it to heat in a much shorter time than other nanomaterials [171]. This phenomenon raises the temperature in the surrounding area, in which the temperature value degree is determined mainly by laser power and the time of irradiation. Furthermore, the shape and size of AuNPs play an essential role in PTT [172]. Halas et al. were the first to report that Au nanoshells might be used as PTT agents. Cells was cultured with PEGylated Au nanoshells while being exposed to an 820 nm laser. After 7 min, considerable cell death was seen in the area of irradiation [173]. Au nanoshells have also been shown to be efficient tumor therapy agents in vivo [174,175]. Additionally, Rastinehad et al. conducted a clinical study that demonstrated that laser treatment using nanoshells (Au coated silica) for prostate cancer successfully achieved an around 94% cure rate in humans, demonstrating the significant potential of AuNPs for cancer therapy [176]. Mohammed et al. described the creation of spherical Au-coated Fe_3_O_4_ as a PTT agent for the eradication of breast cancer cells (MCF 7). Following photothermal therapy for 10 min, MCF 7 cells showed a significant cell reduction of about 73.9% [177].

Among the many geometries of AuNPs, Au nanorods are widely used as PTT agents because of their SPR wavelength and high cell permeability. These properties are a crucial requirement for biological applications. Cheng et al. created spherical AuNP-decorated photolabile diazirine moieties that used in vivo PTT [178]. Under UV irradiation, the produced particles efficiently aggregated at the tumor site, leading to a substantial shift in the peak of SPR towards the NIR region. According to the findings, following AuNP injections over 10 min of irradiation with an 808 nm laser, the localized temperature of the nude mice having 4T1 tumors climbed to 26.7 °C, whereas there was no notable temperature rise in the control tumor. Moreover, when compared with the control, the tumor treated with a dose of AuNPs, followed by laser irradiation, was totally destroyed, implying that PTT is a viable treatment option. Another type of AuNP is Au nanospheres, which have an optical resonance wavelength ranging from 500 to 600 nm depending on size and cover the visible to near-infrared region [171]. It is important to note that the NIR area is advantageous for medical applications since tissues absorb fewer electromagnetic waves within this range [179]. In addition to Au nanospheres being efficient as PTT agents, they can be effective nanocarriers for other chemicals such as metal NPs or cell-labeling agents or, owing to their unique shape, have the potential to be used in theranostics [180,181]. A full investigation of the various shapes of AuNPs used in PTT has been published [179]. Surface ligands play a vital role in tumor targeting and enhancing blood circulation time; nevertheless, they may or may not influence the thermal behavior of AuNPs. Because of the success of this strategy, PTT can be used alone or in conjunction with other approaches, such as chemotherapy, to treat cancer tumors and improve therapeutic efficacy. Numerous other excellent studies have also confirmed that AuNPs, including nanoplates, nanoprisms and nanoparticles, have significant potential for use in PTT cancer treatment, particularly when surface functionalization is used [182,183]. The inclusion of the active target ligands improves the accumulation of the PTT agents in tumor tissue, resulting in the selective killing of cancer cells. AuNPs with strong light-penetrating and conversion efficacy, as well as good compatibility, may be ideal candidates for PTT. Despite the fact that PTT is at the vanguard of cancer treatment, its precise mechanism is still unknown. The key point of contention in current studies is necrotic or apoptotic cell death. Many parameters, such as intensity, particle shape, laser irradiation time, and particle-targeting techniques, all influence the manner in which cells die. As a result, more extensive research is required to determine the essential mechanism of PTT.

### 9.2. Photodynamic Therapy (PDT)

PDT is another interesting cancer therapy technique used in the presence of light-sensitizing agents. PDT is a new cancer treatment technique [184]. It is focused on the use of a photosensitizer that can become excited after being exposed to light. Because of the energy transfer to the environment, reactive oxygen species (ROS) are produced following light irradiation [185]. Clinical photosensitizing compounds, such as phthalocyanines and porphyrins, are typically hydrophobic and thus cannot easily penetrate cells due to lipid barriers. As a result, they require a suitable carrier that can reach cancer cells without modifying the agent. In fact, nanoparticles are used as a carrier for the delivery of photosensitizer drugs in PDT research [186]. Furthermore, the nanoparticle binding to photosensitizing compounds might increase ROS production [187,188]. According to several studies, AuNPs coupled with photosensitizer agents can increase the effectiveness of PDT [189,190,191].

**Figure 9:**
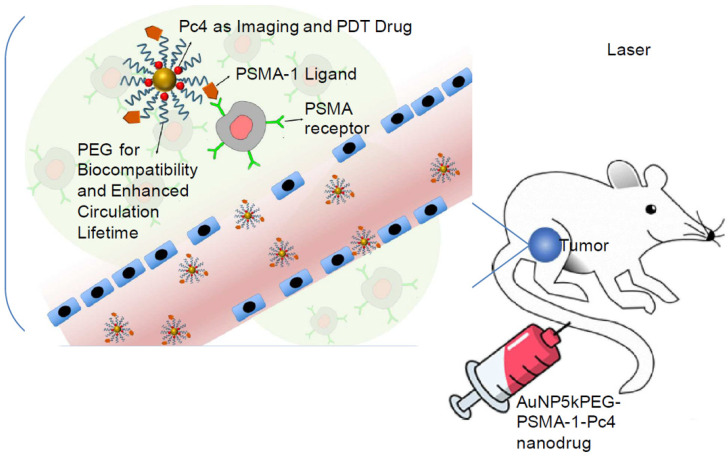
PDT of prostate cancer using AuNPs in a mouse model. Graphical illustration of AuNPs carrying the prostate-specific membrane antigen [191]. Copyright American Chemical Society 2018.

In a mouse model, Burda et al. [186] employed PEGylated coated AuNPs to deliver hydrophobic drugs for PDT. This form of delivery significantly shortened the drug-delivery time as well as improved drug transport to the tumor. Cheng et al. [192] investigated the drug-delivery method and pharmacokinetics of a system containing noncovalent conjugates of the PDT cancer medication with AuNPs. The transport of hydrophobic medicines into tumors in mice by passive build-up of AuNPs resulted in a deep penetration and rapid release into the tumor tissue. Burda et al. [193] covalently linked the photo precursor to AuNPs to produce the photodynamic therapy drug using laser irradiation (660 nm). This enabled the controlled release of drugs, which could increase drug accumulation in the targeted region. When irradiated with light, these nanoparticles successfully deliver Pc 4 to the prostate cancer cells, allowing the tumor to be seen and eventually eliminated (Figure 9). A design like this can help guide surgical therapy and postoperative intervention. Pérez et al. [194] synthesized and integrated a new photosensitizer onto AuNPs via dissymmetric porphyrin derivatives. The photosensitizer-loaded colloidal AuNPs increased the photosensitizer’s activity and water solubility. These studies showed that AuNPs may efficiently overcome photosensitizer hydrophobicity as well as deliver them to tumor locations. Because PTT and PDT have similar mechanisms, most recent research has focused on the combination of PTT and PDT in cancer treatment. In terms of the deep penetration of NIR light, the combination of PDT and PTT can maximize the anticancer efficiency of AuNPs, particularly for deep tumors. At the moment, light and AuNPs are being used to cure cancer cells, and both ROS and hyperthermia are being produced, which can have unanticipated results.

### 9.3. Radiotherapy

**Figure 10:**
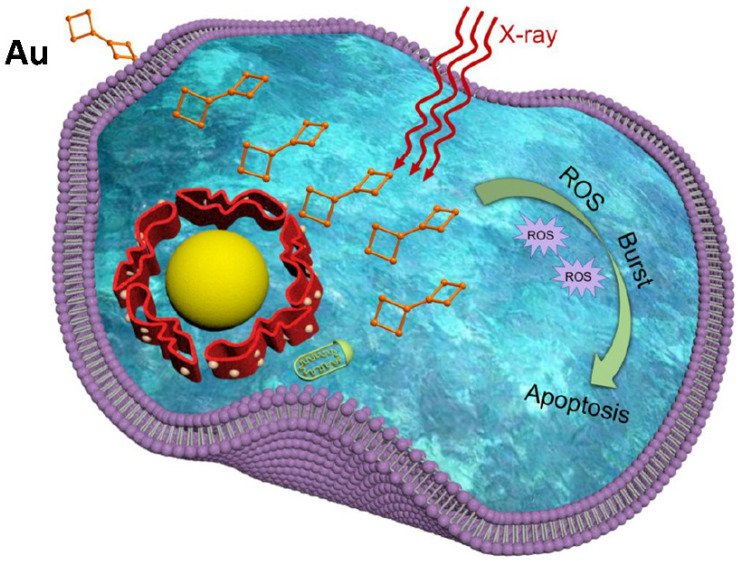
Schematic illustration of the AuNPs for cancer radiotherapy via ROS burst [199]. Copyright American Chemical Society 2019.

**Figure 11:**
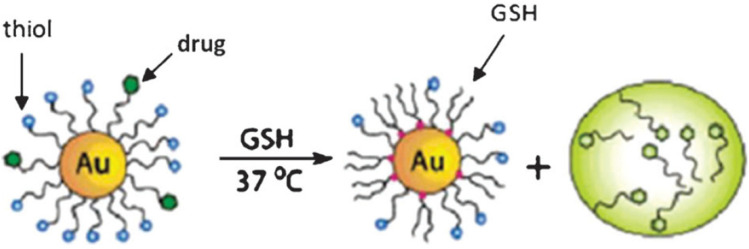
The exchange of intracellular ligands between glutathione and AuNP-coordinated thiolates [205]. Copyright Elsevier 2010.

In clinics, radiotherapy is still the most commonly used cancer treatment technique. The main objective for radiotherapy is to use X-rays with high-energy radiation to shrink tumors and eradicate cancer cells. One of the most difficult aspects of radiotherapy is that X-ray radiation causes collateral harm to neighboring healthy cells (Figure 10). One approach to addressing radiotherapy concerns is to develop potential targeted radiosensitizers that can locally increase radiation damage to tumors while minimizing the harm to proximal tissues. Due to their distinctive photoelectric effects and significant X-ray absorption, AuNPs are one of the most investigated components in this field. The incident electromagnetic waves can interact with AuNPs, resulting in secondary electron emissions. These electrons can cause harm to the inner organs of cell-like mitochondria through direct interaction. Due to the fine Au, AuNP-based materials were regarded as the most appealing radiosensitizers of cancer radiotherapy [195]. Zhang et al. [196,197,198] were the first to bring AuNPs into the area of cancer radiation. A number of atomically exact glutathione-AuNPs, such as Au_25_, Au _29_43_ and Au _10_12_, were discovered to concentrate at the tumor site, particularly due to permeability and retention, and hence have a high radiosensitizing impact in cancer radiotherapy. The thiol/thiolate exchange reactions between the intracellular GSH and thiolate AuNP ligands allow the thiolated drug to be delivered (Figure 11).

AuNPs have also demonstrated rapid renal clearance, resulting in good biocompatibility inside the body. Furthermore, by depleting intracellular glutathione levels with histidine-template AuNPs, an improved cancer radiation method has been devised using both external and internal controls [200]. In vivo investigations have indicated that the glutathione-depleting method is effective for tumor radiation. Broekgaarden et al. [201] discovered that AuNPs modified with glutathione are more appropriate for radiotherapy enhancement through rigorous surface chemistry experiments. Jia et al. [199] revealed in another study that structurally designed levonorgestrel-capped AuNPs with strong fluorescence may be used as a nano-radio-sensitizers for increased cancer irradiation. The AuNPs enhanced intracellular ROS generation in response to X-ray exposure, leading to irreversible death. Furthermore, AuNPs modified with different targeting molecules, including folic acid, peptides and oligonucleotide aptamers, were employed as nano-radio-sensitizers for improved cancer radiotherapy [202,203,204].

### 9.4. Chemotherapy

**Figure 12:**
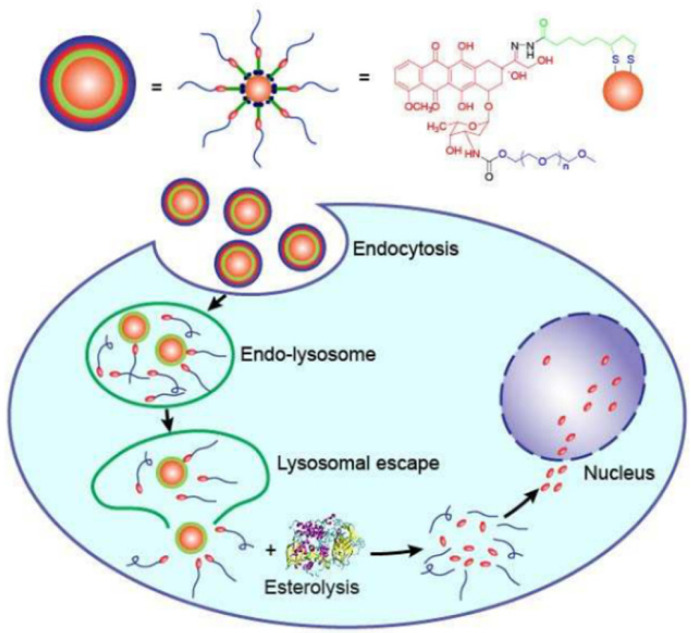
AuNPs are used in cancer treatment. The production and drug release mechanism of DOX-conjugated AuNPs is depicted schematically. The scheme for producing DOX-conjugated AuNPs is shown in the upper half. The lower part depicts the release of the DOX-conjugated AuNPs after endocytosis [220]. Copyright American Chemical Society 2017.

Cancer chemotherapy relies heavily on the targeted administration and bioavailability of chemotherapeutics. Targeted drug delivery using nanomedicine carriers is widely regarded as the most effective way to overcome the poor targeting, need for high dosages and relatively low bioavailability of free medications in conventional medicine [206]. AuNPs have high potential for the targeted administration and regulated release of anticancer medicines due to their good biocompatibility and ease of surface modification (Figure 12). Wang et al. discovered that bovine serum albumin (BSA)-AuNPs loaded with the humanized monoclonal antibody Herceptin can be used for selective targeting and nuclear localization in ErbB2-overexpressing breast cancer [207]. The Herceptin-loaded AuNPs might overcome the endolysosomal route and penetrate the nucleus of cancer cells, enhancing Herceptin’s therapeutic impact. Gu et al. [208] also used functionalized protein-capped AuNPs as cancer drug carriers. The methionine-decorated AuNPs efficiently loaded doxorubicin (DOX) to produce a prodrug for increasing anticancer activity and cancer affinity. To further selectively target tumor and the endothelial cells, iRGD-coupled response bovine serum albumin-AuNPs nanogels have also been employed for targeted DOX delivery [209]. The nanogels’ thermo- and pH-responsive properties may allow for the regulated release of DOX. Bovine serum albumin-AuNPs can also be employed for gemcitabine and DOX co-delivery when conjugated with mesoporous silica nanospheres [210]. Likewise, maytansine analogue DM1-loaded AuNPs have been demonstrated to considerably improve DM1’s therapeutic efficacy against hepatocellular cancer [211]. Furthermore, Chen et al. [212] exploited dual-targeted Apt@cRGD@AuNPs as targeting carriers to enable nuclear-targeted DOX delivery. The DOX bomb has been shown to significantly enhance cancer affinity, antitumor behavior and tumor penetration in many cancer cells and tumor xenograft models. Graphene-assembled AuNPs can also be employed for DOX delivery [213]. By promoting karyopyknosis, the produced nanocomposites might synergistically increase anticancer efficacy. Dendrimer-encapsulated AuNPs can be used to load anticancer medicines such as DOX, thiolated cisplatin, captopril and 6-mercaptopurine via Au-S bonding [214]. In the presence of glutathione, the loaded medicines displayed an “off–on” release pattern. Intracellular glutathione performs a scissor function, potentially causing the release of loaded chemotherapeutics within AuNPs, resulting in cell death. Because of folate receptor-mediated endocytosis, various decorated AuNPs can be employed as nanocarriers to deliver anticancer medicines such as paclitaxel (PTX), DOX, cisplatin and camptothecin (CPT) to cancer cells via folic acid functionalization [215,216,217,218]. Arunakaran et al. [219] investigated the effects of AuNPs with a diameter of about 3 nm coupled with quercetin on breast cancer cell lines (MCF 7). The findings suggest that quercetin-conjugated AuNPs are more powerful than free quercetin and might be employed for targeted medication delivery with improved therapeutic efficacy in the breast cancer treatment. The NPs suppressed the phosphorylation of the epidermal growth factor receptor (EGFR) as well as molecule activity in MCF 7. These AuNP-based drug delivery systems give evidence for AuNPs’ potential use in cancer treatment.

### 9.5. Drug Delivery

The potential of nanoparticles for us as a drug delivery system to carry drugs is 10 to 100 times greater than the molecular administration of the drug to tumors and so can improve therapeutic and diagnostic methods [221]. Furthermore, because of less uptake through the reticula-endothelial system, the drug circulation period within nanoparticles can be prolonged, and it can accelerate drug uptake by tumor cells [184,222]. Because of properties such as high affinity, nontoxicity and biocompatibility, the surface of AuNPs can be employed for active tumor targeting with biomarkers, antibodies and ligands capable of selective binding to tumors. Cytotoxic drug delivery at specific sites can enhance diagnosis and treatment whilst reducing undesirable side effects [185,223]. Various studies have used AuNPs to administer anticancer medications such as paclitaxel [224], tamoxifen [225], methotrexate [226] and platinum-based drugs, including oxaliplatin and cisplatin, to promote therapeutic efficiency [227,228].

Passive targeting is an easy mechanism for delivering anticancer drugs using AuNPs, and is based solely on particle size. In this method, NPs accumulate in the tumor location, utilizing the effect of increased permeability and retention (RES). Furthermore, in order to develop an efficient delivery system, combining passive or active targeting would be highly useful [229]. The core challenge that AuNPs face is being undetected by the RES and immune system, which has a considerable impact on their capacity to reach the designated tissue and the speed of blood circulation. According to various studies, bare AuNPs have a fast clearance rate, which inhibits their successful delivery [112,230]. PEG is a commonly employed polymer that, due to its excellent hydrophilicity, can increase the circulation period of NPs in the bloodstream [111]. Lipka et al. [231] produced AuNPs with a diameter of 5 nm followed by modification with PEG1000, which significantly enhanced the blood circulation time compared to those treated with PEG750. To ensure optimal cellular absorption in cancer therapy, several forms of AuNPs with a large size range should be investigated. For example, the absorption of Au nanorods by tumor cells is higher than for other shapes because of their structure and increased permeability [232]. Methotrexate is an anticancer medicine widely used as a chemotherapeutic agent in treating prostate, breast and lung cancers. However, it has recently been demonstrated that impairing the drug import of cells and then increasing drug export from them results in robust cell resistance to methotrexate [233]. As a result, developing a new way to address this issue appears crucial. Thirteen-nanometer colloidal AuNPs coupled with methotrexate via a carboxylic group (COOH) demonstrated a greater anticancer effect on many tumor cell lines than free methotrexate [226]. This could be attributed to the increased methotrexate accumulation, which contributes to high drug concentrations in tumor cells treated with methotrexate–AuNP treated with free drugs. Cancer cells that contain folate receptors can identify and absorb folic acid-modified AuNPs. Rizk et al. investigated the therapeutic efficacy of methotrexate-loaded sphere AuNPs (100 nm) functionalized with folic acid as an MCF 7 targeting agent [234]. Compared to the free drugs, the results indicated greater cellular absorption by a metastatic human MCF 7 type and decreased cytotoxicity against normal cells. Heo et al. [235] studied complex paclitaxel-loaded AuNPs functionalized with rhodamin B, biotin, PEG and cyclodextrin to improve blood circulation and water solubility, used as the fluorescent probe and targeting ligand. They tested cell cytotoxicity against several types of cancer cells: NIH3T3, MG63 and A542. The developed compound demonstrated a higher cellular uptake and anticancer activity than the free drug and, in general, can be used as a platform for current drug-delivery agents. Moreover, the produced nanoparticles performed well as detecting agents in a variety of diagnostic systems, including cell viability studies, confocal laser scanning microscopy and fluorescence-activated cell sorting. pH-sensitive DOX-loaded AuNPs were used for cancer theranostics in a recent work [236]. The maximum amount of drug released was observed under acidic tumor circumstances (in 102 h at about 88% h). Furthermore, when compared to pure DOX, the produced complex demonstrated better anticancer activity and better CT imaging characteristics in vitro and in vivo.

## 10. Current Limitations

AuNPs show promise and can be used in cancer detection and therapy. However, it is critical to address the opposite side of the coin, namely, unforeseen health consequences. Individual investigations of the retention period, biodistribution, efficacy, cytotoxicity, the effect of nanoparticle size on toxicity and physiological response of AuNPs have already been conducted. Many of them, though, appear to contradict one another. The lack of consistent information on the actual impacts of nanoparticles may cause issues and have a harmful influence on human health. While the challenges highlighted generally apply to any nanoparticle, the examples provided below are unique to AuNPs. AuNPs’ toxicity to biological systems has long been a source of concern [237]. The size, shape, targeted ligand, surface chemistry and composition of AuNPs all have a significant impact on their toxicity. The surface charge of AuNPs has been shown to influence their toxicity, with positively charged particles being found to be more poisonous than negatively or neutrally charged particles [238]. Other teams discovered no toxicity caused by negatively charged AuNPs [239] and no toxicity caused by positively charged particles [240]. This disparity originates from the distinct physiochemical nature of NPs, and there is currently no standardized assay that can be used to determine the toxicity of all nanoparticles.

In addition to toxicity assessment, the size and biodistribution of nanomaterials are important elements to consider. Tang et al. [241] found that smaller AuNPs of about 8 nm coated with reduced glutathione were more toxic to a human hepatic cell line than larger particles of about 37 nm. Rosli et al. [242] found that 50 nm AuNPs were more toxic to breast cancer cells than their 13 and 70 nm counterparts. Connor et al. [243] investigated the cytotoxicity of a range of AuNP sizes on human leukemia cells and observed that none of the sizes were damaging to cellular function.

## 11. Challenges and Future Perspectives

Physical and chemical procedures are commonly used for the synthesis of AuNPs and their rapidly expanding prospective uses in medical areas should be studied. However, the use of toxic and expensive chemicals, as well as complicated apparatus, has limited their economic potential. As a result of the difficulties associated with the traditional methods of synthesis, researchers have been encouraged to develop green chemistry-based and cost-effective procedures for the synthesis of AuNPs in order to meet the growing industrial demands for AuNPs. Because of its renewability and eco-friendliness, the phytosynthesis of AuNPs is recognized as a critical method. However, the use of commercially valuable foods and plants as reducing and stabilizing agents has a negative effect on the function of synthetic techniques. As a result, it is critical to shift the attention to investigating the reduction and stabilization potential of noncommercially valuable plants, notably biowastes, for the synthesis of AuNPs in order to improve the efficacy of the biosynthetic process. Plant biowastes contain bioactive chemicals that act as reducing and stabilizing agents during the synthesis of AuNPs. However, because of the complexity of plant constituents, it is impossible to exactly determine the phytochemicals involved in AuNP production. Furthermore, estimating the decreased potential for each element of the plant extracts is more difficult. As a result, it is extremely important to utilize qualitative and quantitative methodologies for the exact measurement of bioactive chemicals involved in gold ion reduction and AuNP stabilization. This would be extremely advantageous in creating AuNPs with appropriate physicochemical properties for future applications. It would also provide a deeper understanding of the synthesis reaction and the specific reduction and stabilization mechanism, which have yet to be completely investigated. The synthesis conditions have a significant impact on the physicochemical properties of biosynthesized AuNPs. As a result, determining the effect of process factors on the size and shape of phytosynthesized AuNPs is very interesting. The examination of synthesis conditions on developing the surface features of AuNPs would aid in the production of nanoparticles with the desired sizes and shapes, which would be extremely useful in determining their prospective uses.

## 12. Conclusions and Outlook

As this study has demonstrated, there is currently a great deal of optimism and excitement regarding the use of AuNPs for a wide range of medical applications. There is also a definite need to thoroughly investigate their long-term effects on the environment and human health. We predict that, as new nanoparticle systems demonstrate potential in research, these materials will be examined for clinical applications in the near future. We discussed the benefits and biomedical uses of monodisperse AuNPs, with a focus on theranostic applications. Size is one of the most important physical variables of AuNPs, and it has a direct impact on their features, such as toxicity and biocompatibility. A greater understanding of the characteristics of monodisperse AuNPs will facilitate the design of AuNP-based nanoplatforms, allowing their medical applications to be expanded. Surface functionalization and modification are critical in the development of monodisperse AuNPs. Monodisperse AuNPs with outstanding electrical and optical properties are currently being employed as contrast agents in optical imaging, photoacoustic imaging, fluorescence imaging, CT and MRI. Importantly, monodisperse AuNPs can specifically deliver agents and target tumor tissues for chemotherapy, photodynamic treatment, and other treatments to improve the efficiency of killing cancer cells. Although the current research findings on monodisperse AuNPs in medical applications are intriguing, there are still challenges that require further investigation. First, more research on the combination of diagnosis and treatment related to the physical features of AuPNs is required. Second, as a universal difficulty in cancer therapy, patient differences complicate the optimization of AuNPs for cancer therapy, and further efforts are required to overcome this barrier. Nevertheless, the ease with which monodisperse AuNPs can be functionalized offers many possibilities for customized therapy. Given the effectiveness of monodisperse AuNPs in imaging and treatment for cancer, the implementation of appropriate techniques to overcome barriers and achieve AuNP-dependent cancer cell death looks very promising. Considering all of this information, we feel that monodisperse AuNPs provide unique opportunities to transform fundamental research findings into clinical applications.

## Figures and Tables

**Table 1:** Different methods of synthesizing AuNPs.

Nanoparticle	Method	Size (nm)	Shape	Application	Ref
AuNPs	Turkevich	3.5	Spherical	……….	[49]
AuNPs	Seed-mediated	30–150	Spherical	……….	[51]
AuNPs	Laser irradiation	24	Spherical	……….	[52]
AuNPs	γ-irradiation	5–40	Semi-spherical	……….	[59]
AuNRs	Chemical reduction	……….	rod	……….	[63]
AuNPs	Reduction by glutamic acid	40	Spherical	Bioconjugates	[64]
AuNPs	Brust	1–3	Semi-spherical	……….	[66]
AuNPs	Reduction	180	Decahedral	……….	[69]
AuNPs	Green	10	Spherical, triangular and hexagonal	……….	[70]
AuNPs	Sonochemical	22	Spherical	……….	[88]
AuNPs	Laser ablation	49	Spherical	……….	[88]
AuNPs	Sonochemical	18.5	Spherical	Computed tomography	[5]
AuNPs	Sonochemical	13.6, 18.6 and 22.3	Spherical	Catalysis	[89]

## Data Availability

Not applicable.

## Abbreviation

AuNPs	Gold nanoparticles
NPs	nanoparticles
SPR	surface plasmon resonance
NIR	near-infrared
CT	computed tomography
PAI	photoacoustic imaging
MRI	magnetic resonance imaging
FWHM	full width half maximum
SB	sodium borohydride
SC	sodium citrate
NBP	nanobipyramid
NRS	nanorod shell
TEM	Transmission electron microscopy
SEM	Scanning electron microscope
SERS	Surface-Enhanced Raman Resonance
FA	folic acid
MBA	4-mercaptobenzoic
BSA	bovine serum albumin
RBSA	reduced bovine serum albumin
ALP	alkaline phosphatase
SIO_2_	Silicon dioxide
Ag	silver
DOX	doxorubicin
PSS	poly sodium 4-styrenesulfonate
UV	Ultraviolet
HAuCl_4_	gold acid
HRTEM	high resolution Transmission electron microscopy
SAED	selected area electron diffraction
NaBH_4_	Sodium borohydride
OCT	optical coherence tomography
DNA	Deoxyribonucleic acid
CTAB	hexadecyl trimethyl ammonium bromide
siRNA	small interfering Ribonucleic acid
HER	Herceptin
HER2	human epidermal growth factor receptor 2
DFM	dimethylformamide
FI	fluorescence imaging
EPR	enhanced permeability and retention
GSH	glutathione
CAL-27	human tongue squamous cell carcinoma
PSMA	prostate specific membrane antigen
Gd	gadolinium
T_2_-relaxation	spin–spin relaxation
NMR	nuclear magnetic resonance
DTPA	diethylenetriamine pentaacetate
LA	lactobionic acid
EDC	1-ethyl-3-(3-dimethylaminopropyl)carbodiimide
NHS	n-hydroxysuccinimide
NBP	nanobipyramid
PET	positron emission tomography
^64^Cu	copper-64
RGD	tripeptide arg-gly asp
PPT	photothermal therapy
PDT	photodynamic therapy
Fe_3_O_4_	iron oxide
MCF 7	breast cancer cell line
4T1	breast cancer cell line
ROS	reactive oxygen species
ErbB2	receptor tyrosine kinase 2
DM1	emtansine
PTX	paclitaxel
CPT	camptothecin
EGFR	epidermal growth factor receptor
RES	permeability and retention;
COOH	carboxylic group
NIH3T3	fibroblast cell line
MG63	hypotriploid human cell line
A542	epithelial cell
